# Evolutionary perspectives on human infectious diseases: Challenges, advances, and promises

**DOI:** 10.1111/eva.12586

**Published:** 2018-04-09

**Authors:** Pierre Echaubard, James W. Rudge, Thierry Lefevre

**Affiliations:** ^1^ Global Health Asia Institute Faculty of Public Health Mahidol University Bangkok Thailand; ^2^ Department of Biology Laurentian University Sudbury ON Canada; ^3^ Department of Global Health and Development London School of Hygiene and Tropical Medicine London UK; ^4^ Faculty of Public Health Mahidol University Bangkok Thailand; ^5^ Institut de Recherche en Sciences de la Santé (IRSS) Bobo Dioulasso Burkina Faso; ^6^ MIVEGEC, IRD, CNRS University. Montpellier Montpellier France

## 1 INTRODUCTION

Modern biomedicine has contributed remarkably to the reduction of infectious diseases worldwide, including the eradication of smallpox and the control of common childhood diseases (e.g., polio, measles, rubella) that once claimed millions of lives and caused suffering of tens of millions. This has been made possible through improved diagnostics, surveillance, therapeutics, vaccines, and an associated health system infrastructure. These achievements can be largely credited to advances in biomedical sciences and their application in the 20th century.

The spectacular advances in the biomedical sciences were in turn a consequence of the emergence and widespread acceptance of the biomedical paradigm, in which the discovery of microbes and establishment of ‘germ theory’ had played a central role. By the 1960s, a sense began to prevail that infectious diseases had been conquered, or were at least conquerable in the case of malaria, dengue, and other vector‐borne diseases (VBD) that appeared to be “in retreat.”

Yet, by the 1980s, with the appearance of the HIV/AIDS pandemic and followed by new antimicrobial‐resistant strains of bacterial pathogens, confidence began to erode. Major setbacks also increasingly became apparent in the efforts to control VBDs particularly with the resurgence of malaria and dengue worldwide (WHO, 2016). An increasing number of historically localized or otherwise geographically confined VBDs began to spread and even jump continents, with the arrival of West Nile virus and Zika virus in the Americas being the most prominent of many examples. Such pathogens, identified decades ago in their native habitat and hosts as part of natural history studies, were long considered to be of relatively little public health concern or apparent impact (Lederberg, Shope, & Oaks, 1992; Packard, 2007).

Human population growth and anthropogenic environmental changes, accelerating with an unprecedented intensity and scale particularly since the mid‐1900s, are increasingly recognized as underlying much of the emerging infectious diseases (EID) problem (Myers & Patz, 2009; Myers et al., 2013; Whitmee et al., 2015). These disease emergence drivers emblematic of the era of modern development can be seen as consequences of modern medical and hygiene interventions introduced a century earlier. Together, they radically modified (and continue to alter) landscapes and ecosystems worldwide producing what has become a continual state of social, ecological, and evolutionary imbalance (McNeill, 1976). Among the environmental changes, global climate change has become widely accepted among experts as a significant contributor to these imbalances that will increasingly influence infectious diseases transmission, yet in ways that are difficult to predict (Altizer, Ostfeld, Johnson, Kutz, & Harvell, 2013; Lafferty, 2009; McMichael & Wilcox, 2009).

The complexity of factors and processes underlying infectious diseases and their transmission go well beyond the scope and analytic resolution of biomedicine (and conventional biomedical training), requiring a complementary framing approach capable of acknowledging and assessing cross‐scale influences, context dependency, and the constant ‘arms race’ between co‐evolving organisms (Van Valen, 1973). Evolutionary biology provides these scientific foundations to help refine our understanding of not only the meaning of health and disease (Stearns & Koella, 2008) from the standpoint of adaptation (or maladaptation), but also improve our understanding of the mechanisms underlying infectious disease transmission dynamics within social‐ecological systems (Horwitz & Wilcox, 2005; Wilcox & Echaubard, 2016), context‐dependent virulence and more effective treatment and control strategies (Echaubard, Sripa, Mallory, & Wilcox, 2016; Nesse, 2008; Restif, 2009). As such, evolutionary biology as a framing approach and methodological toolkit is a needed component of integrated disease control and prevention (Allegranzi et al., 2017) and sustainable health development aligning with the recently adopted sustainable development goals (SDGs), as part of the 2030 Agenda for Sustainable Development (Carroll et al., 2014).

This special issue is an attempt to present an up‐to‐date appraisal of the challenges, current advances, and promising research avenues where evolutionary principles and their ecological corollaries can be applied in research as a basis for human infectious disease interventions. Accordingly, the contributions published in this special issue together present an illustration of the diverse benefits of combining biomedical and public health perspectives with evolutionary causation in the context of infectious diseases for major infectious agents (Table 1).

**Table 1:** Summary of public health data, regular diagnostic and treatment, main control strategy as well as challenges for control of the disease investigated in this special issue

Disease	Public health situation	Diagnostic & treatment	Control strategy	Challenges for control
Malaria^a^	212 million new cases of malaria worldwide in 2015 The WHO African Region accounted for most global cases of malaria (90%), followed by the South‐East Asia (SEA) Region (7%) and the Eastern Mediterranean Region (2%). In 2015, there were an estimated 429 000 malaria deaths worldwide (Africa 92%, SEA Region 6%, Mediterranean Region 2%). Between 2010 and 2015, malaria incidence rates fell by 21% globally and in the African Region. During this same period, malaria mortality rates fell by an estimated 29% globally and by 31% in the African Region. Since 2010, the malaria mortality rate declined by 58% in the Western Pacific Region, by 46% in the SEA Region, by 37% in the Region of the Americas, and by 6% in the Eastern Mediterranean Region. In 2015, the European Region was malaria free In 2015, malaria killed an estimated 303,000 under‐fives globally, including 292,000 in the African Region. Between 2010 and 2015, the malaria mortality rate among children under 5 fell by an estimated 35%. Nevertheless, malaria remains a major killer of under‐fives, claiming the life of 1 child every 2 min.	WHO recommends diagnostic testing for all people with suspected malaria before treatment is administered. Rapid diagnostic testing (RDTs), introduced widely over the past decade, has made it easier to swiftly distinguish between malarial and nonmalarial fevers, enabling timely and appropriate treatment. In 2015, approximately half (51%) of children with a fever who sought care at a public health facility in 22 African countries received a malaria diagnostic test compared to 29% in 2010. Artemisinin‐based combination therapies (ACTs) are highly effective against *P. falciparum*, the most prevalent and lethal malaria parasite affecting humans. Globally, the number of ACT treatment courses procured from manufacturers increased from 187 million in 2010 to a peak of 393 million in 2013, but subsequently fell to 311 million in 2015.	Vector control is the main way to prevent and reduce malaria transmission. Two forms of vector control are effective in a wide range of circumstances: insecticide‐treated mosquito nets (ITNs) and indoor residual spraying (IRS). Over the last 5 years, the use of treated nets in the region has increased significantly: in 2015, an estimated 53% of the population at risk slept under a treated net compared to 30% in 2010. In 2015, 106 million people globally were protected by IRS, including 49 million people in Africa. The proportion of the population at risk of malaria protected by IRS declined from a peak of 5.7% globally in 2010 to 3.1% in 2015.	Artemisinin resistance mostly in the Mekong subregion, at Thailand–Myanmar and Thailand–Cambodia borders. Mosquito resistance to insecticides Composition of *Anopheles* species complexes have important implications for control strategies Transboundary labor migration Intensive agricultural cropping systems, such as fruit orchards, increase the likelihood of insecticide resistance development in malaria mosquitoes
Dengue^b^	One recent estimate indicates 390 million dengue infections per year (95% credible interval 284–528 million), of which 96 million (67–136 million) manifest clinically The prevalence of dengue is estimated at 3.9 billion people, in 128 countries, at risk of infection with dengue viruses The number of cases reported increased from 2.2 million in 2010 to 3.2 million in 2015. Before 1970, only 9 countries had experienced severe dengue epidemics. The disease is now endemic in more than 100 countries in the WHO regions of Africa, the Americas, the Eastern Mediterranean, SEA, and the Western Pacific. Cases across the Americas, South‐East Asia, and Western Pacific exceeded 1.2 million in 2008 and over 3.2 million in 2015. Recently, the number of reported cases has continued to increase. In 2015, 2.35 million cases of dengue were reported in the Americas alone, of which 10,200 cases were diagnosed as severe dengue causing 1181 deaths. An estimated 500,000 people with severe dengue require hospitalization each year, and about 2.5% of those affected die.	Dengue fever is a severe, flu‐like illness that affects infants, young children, and adults Dengue should be suspected when a high fever is accompanied by severe headache, pain behind the eyes, muscle and joint pains, nausea, vomiting, swollen glands or rash. Severe dengue is a potentially deadly complication due to plasma leaking, fluid accumulation, severe bleeding, or organ impairment. There is no specific treatment for dengue fever. Maintenance of the patient's body fluid volume is critical to severe dengue care. In late 2015 and early 2016, the first dengue vaccine, Dengvaxia (CYD‐TDV) by Sanofi Pasteur, was registered in several countries for use in individuals 9–45 years of age living in endemic areas. WHO recommends that countries should consider introduction of the dengue vaccine CYD‐TDV only in geographical settings where epidemiological data indicate a high burden of disease.	At present, the main method to control or prevent the transmission of dengue virus is to combat vector mosquitoes through: Preventing mosquitoes from accessing egg‐laying habitats by environmental management and modification Disposing of solid waste and covering, emptying, and cleaning of domestic water storage containers on a weekly basis Applying appropriate insecticides to water storage outdoor containers and environmental management Using of personal household protection such as window screens, long‐sleeved clothes, insecticide‐treated materials, coils, and vaporizers; Improving community participation and mobilization for sustained vector control	Increasing population movement, globalization of trade and urbanization without adequate measures to prevent vector breeding Rubber plantations expansion Mosquito vector and pathogen adaptation
Influenza (zoonotic)	Humans can be infected with avian, swine, and other zoonotic influenza viruses, such as avian influenza virus subtypes A(H5N1), A(H7N9), and A(H9N2) and swine influenza virus subtypes A(H1N1), A(H1N2), and A(H3N2). The majority of human cases of influenza A (H5N1) and A(H7N9) virus infection have been associated with direct or indirect contact with infected live or dead poultry. Controlling the disease in the animal source is critical to decrease risk to humans. Aquatic birds are the primary natural reservoir for most subtypes of influenza A viruses. Most cause asymptomatic or mild infection in birds, where the range of symptoms depends on the virus properties. Viruses that cause severe disease in poultry and result in high death rates are called highly pathogenic avian influenza (HPAI). Viruses that cause mild disease in poultry are called low‐pathogenic avian influenza (LPAI).	Avian, swine, and other zoonotic influenza virus infections in humans may cause disease ranging from mild upper respiratory tract infection (fever and cough), early sputum production, and rapid progression to severe pneumonia, sepsis with shock, acute respiratory distress syndrome and even death. Laboratory tests are required to diagnose human infection with zoonotic influenza. Rapid influenza diagnostic tests (RIDTs) have lower sensitivity compared to PCR and their reliability depends largely on the conditions under which they are used. Evidence suggests that some antiviral drugs, notably *neuraminidase inhibitor* (oseltamivir, zanamivir), can reduce the duration of viral replication and improve prospects of survival; however, ongoing clinical studies are needed. In suspected and confirmed cases, neuraminidase inhibitors should be prescribed as soon as possible to maximize therapeutic benefits.	Influenza viruses, with the vast silent reservoir in aquatic birds, are impossible to eradicate. To minimize public health risk, quality surveillance in both animal and human populations, thorough investigation of every human infection and risk‐based pandemic planning are essential. Apart from antiviral treatment, the public health management includes personal protective measures like regular hand washing, good respiratory hygiene and early self‐isolation of those feeling unwell, feverish and having other symptoms of influenza. Pre‐exposure or postexposure prophylaxis with antivirals is possible but depends on several factors, for example, individual factors, type of exposure, and risk associated with the exposure.	International movement of strains Genetic reassortments Emergence of oseltamivir resistance has been reported. Most recent A(H5) and A(H7N9) viruses are resistant to adamantane antiviral drugs (e.g., amantadine and rimantadine) and are therefore not recommended for monotherapy.
Chagas disease^c^	Chagas disease, also known as American trypanosomiasis, is a potentially life‐threatening illness caused by the protozoan parasite *Trypanosoma cruzi (T. cruzi)*. About 6 million to 7 million people worldwide are estimated to be infected with *Trypansosoma cruzi*. Chagas disease is found mainly in endemic areas of 21 Latin American countries, where it is mostly vector‐borne transmitted to humans by contact with feces or urine of triatomine bugs Chagas disease occurs principally in the continental part of Latin America and not in the Caribbean isles. In the past decades, however, it has been increasingly detected in the United States of America, Canada, and many European and some Western Pacific countries. This is due mainly to population mobility between Latin America and the rest of the world.	Chagas disease can be treated with benznidazole and also nifurtimox. Both medicines are almost 100% effective in curing the disease if given soon after infection at the onset of the acute phase including the cases of congenital transmission. The efficacy of both diminishes, however, the longer a person has been infected. The potential benefits of medication in preventing or delaying the development of Chagas disease should be weighed against the long duration of treatment (up to 2 months) and possible adverse reactions (occurring in up to 40% of treated patients). The cost of treatment for Chagas disease remains substantial. In Colombia alone, the annual cost of medical care for all patients with the disease was estimated to be about US 267 million in 2008. Spraying insecticide to control vectors would cost nearly US 5 million annually—less than 2% of the medical care cost. There is no vaccine for Chagas disease.	Vector control is the most effective method of prevention in Latin America. Blood screening is necessary to prevent infection through transfusion and organ transplantation. The control targets are elimination of the transmission and early healthcare access for the infected and ill popultion. Depending on the geographical area, WHO recommends the following approaches to prevention and control: 1 ◦ Spraying of houses and surrounding areas with residual insecticides 2 ◦ House improvements and house cleanliness to prevent vector infestation; 3 ◦ Bednets	Psycho‐social challenges such as stigma related to poverty and emotional fears of being judged leading to low reporting and screening Increasing insecticide resistance of Triatomine vectors and rapid recolonisation of households by vectors after spraying Diagnosis
Schistosomiasis^d^	Schistosomiasis is an acute and chronic parasitic disease caused by blood flukes (trematode worms) of the genus *Schistosoma*. Estimates show that at least 206.4 million people required preventive treatment in 2016. Schistosomiasis transmission has been reported from 78 countries. However, preventive chemotherapy for schistosomiasis, where people and communities are targeted for large‐scale treatment, is only required in 52 endemic countries with moderate‐to‐high transmission. Schistosomiasis is prevalent in tropical and subtropical areas, especially in poor communities without access to safe drinking water and adequate sanitation. It is estimated that at least 91.4% of those requiring treatment for schistosomiasis live in Africa. There are 2 major forms of schistosomiasis—intestinal and urogenital—caused by 5 main species of blood fluke. Schistosomiasis mostly affects poor and rural communities, particularly agricultural and fishing populations. Women doing domestic chores in infested water, such as washing clothes, are also at risk and can develop female genital schistosomiasis.	Schistosomiasis is diagnosed through the detection of parasite eggs in stool or urine specimens. Antibodies and/or antigens detected in blood or urine samples are also indications of infection. For urogenital schistosomiasis, a filtration technique using nylon, paper, or polycarbonate filters is the standard diagnostic technique. Children with *S. haematobium* almost always have microscopic blood in their urine, which can be detected by chemical reagent strips. The eggs of intestinal schistosomiasis can be detected in fecal specimens through a technique using methylene blue‐stained cellophane soaked in glycerin or glass slides, known as the Kato‐Katz technique.	The control of schistosomiasis is based on large‐scale treatment of at‐risk population groups, access to safe water, improved sanitation, hygiene education, and snail control. The WHO strategy for schistosomiasis control focuses on reducing disease through periodic, targeted treatment with praziquantel through the large‐scale treatment (preventive chemotherapy) of affected populations. It involves regular treatment of all at‐risk groups. In a few countries, where there is low transmission, the interruption of the transmission of the disease should be aimed for.	Environmental degradation Dams and large‐scale irrigation project that contribute to snail intermediate host proliferation Animal reservoirs Potential for praziquantel resistance

a WHO (2016).

b Bhatt et al. (2013); Brady et al. (2012).

c Tarleton, Reithinger, Urbina, Kitron, and Gürtler (2007).

d World Health Organization & UNICEF (2004).

## 2 THEMES OF THE SPECIAL ISSUE

A main challenge of mainstreaming evolution into medicine and public health lies in the often very pragmatic and urgent nature of these fields (e.g., outbreak mitigation; Nesse & Stearns, 2008) and the relatively time‐consuming nature of framing questions, interventions, and policies following evolutionary principles, which imply a long‐term preventive vision rather than a short‐term curative domain of action. However, policy‐oriented research and intervention informed by evolutionary biology have the potential to not only effectively mitigate urgent crises but also anticipate, minimize, and respond to the evolution of unwanted epidemiological traits (e.g., antimicrobial resistance). Accordingly, the articles compiled for this special issue present a selected panel of evolutionary applications for infectious disease control, together providing relevant avenues for future research and sustainable disease prevention strategies.

The articles published in this special issue are organized based on their underlying evolutionary innovation and public health implications and not necessarily on the disease study systems the authors are using to address their research questions. A range of disease systems, from macroparasites to microparasites (sensu Anderson & May, 1981), are examined through modeling, experimental and observational research designs as well as describing novel framing approaches.

As an opening paper, Kosoy and Kosoy (2017) explore the multiple dimensions and related complexity of host–pathogens interactions revealed by the novel genetic and genomic data, along with extensive environmental parameters being acquired using newly developed computational tools. They highlight the need for flexibility in studying natural systems of zoonotic pathogens with respect to how we choose perspectives within a continuum between unrestricted diversity of related parameters and well‐defined roles played by infectious agents, potential and actual animal hosts, and environmental variables. They proposed a model of investigation that requires a dynamic shift of perspectives along the simplicity–complexity (‘simplexity’) dimension emphasizing the difficulty to accommodate dual representations of both the subjective nature of investigations of zoonotic pathogens and much more objectively derived information, for example, coded in the genetic structure of DNA or in observing the morphology or behavior of bacteria. This also speaks directly to the issue of “problem framing” alluded to in this introduction where tools and perceptions emerging from biomedical sciences do not necessarily accommodate evolutionary‐based predictions or public health implications.

Arguably the most widespread recognition of the importance of evolutionary biology for public health and medicine is in the context of the emergence of resistance to antimicrobials and insecticides. Sternberg and Thomas (2017) explore the overlaps between understanding and managing insecticide resistance in agriculture and in public health with the aim to identify best practices in resistance mitigation strategies in the context of vector‐borne disease interventions. They argue that the success of insecticide resistance management strategies is strongly dependent on the biological specifics of each system and that the biological, operational, and regulatory differences between agriculture and public health limit the wholesale transfer of knowledge and practices from one system to the other. Nonetheless, the authors argue that there are some valuable insights from agriculture that could assist in advancing the existing Global Plan for Insecticide Resistance Management (IRM). Accordingly, the authors suggest that for IRM strategies to succeed in public health, there needs to be a shift away from choosing vector control tools or strategies based on direct cost, toward factoring in the benefit of preserving susceptibility.

Focusing on Malaria, Huijben and Paaijmans (2017) analyze the evolutionary consequences of the way antimalarial drugs and insecticide‐based interventions are currently implemented, which is leading to resistance and may ultimately lead to control failure. The authors describe how evolutionary principles can be applied to extend the lifespan of current and novel interventions highlighting in particular how understanding fitness costs arising from expressing, utilizing, and maintaining molecular or metabolic pathways of resistance will be essential to mitigate resistance evolution. They continue arguing that similar to insecticide resistance management strategies, large heterogeneity in drug exposure can be created in space (host mosaic) or time (drug rotation) or by deploying different compounds simultaneously (mixed treatment) and that better resistance management is achieved if drugs can be combined that select for alternative allelic versions of the target locus.

Glunt et al. (2017) further discuss the challenges of insecticide resistance in the context of malaria, with a specific focus on pyrethroids impregnated long‐lasting insecticidal bed nets (LLINs) and their efficacy in preventing malaria. About 1 billion LLINs, a major vector control tool, have been distributed in Africa in the last 10 years. During the same period of time resistance to pyrethroids in malaria mosquito vectors has increased significantly. Using a transmission model, the authors show that when LLIN‐related lethal and sublethal effects were accrued over mosquito lifetimes, they greatly reduced the impact of resistance on malaria transmission potential under conditions of high net coverage. However if coverage falls, the epidemiological impact is far more pronounced. Similarly, if the intensity of resistance intensifies, the loss of malaria control increases nonlinearly. The authors argue that their findings help explain why insecticide resistance has not yet led to wide‐scale failure of LLINs, as high distribution coverage is generally in place in most African endemic countries, but reinforce the call for alternative control tools and informed resistance management strategies.

While parasites can evolve classical resistance mechanisms (e.g., efflux pumps), it is also possible that changes in life‐history traits could help parasites evade the effects of treatment. Birget, Greischar, Reece, and Mideo (2017) investigate how the life history of malaria parasites is governed by an intrinsic resource allocation problem where specialized stages are required for transmission, but producing these stages comes at the cost of producing fewer of the forms required for within‐host survival. The underlying rationale is that drug treatment, by design, alters the probability of within‐host survival and so should alter the costs and benefits of investing in transmission. The authors use a within‐host model of malaria infection to predict optimal patterns of investment in transmission in the face of different drug treatment regimens and determine the extent to which alternative patterns of investment can buffer the fitness loss due to drugs. This work emphasizes that in addition to classical resistance mechanisms, drug treatment generates selection for altered parasite life history. It also suggests that understanding how any shifts in life history will alter the efficacy of drugs, as well as any limitations on such shifts, is important for evaluating and predicting the consequences of drug treatment.

While evolutionary principles can help design more sustainable insecticide and antimicrobial resistance mitigation strategies, evaluating the risk of emergence and transmission of vector‐borne diseases also requires knowledge of the genetic and environmental contributions to pathogen transmission traits. In their perspective article, Lefevre et al. (2017) discuss how the associations between malaria parasites transmission traits and their related trade‐offs and constraints could have important implications for understanding the evolution of parasite transmission and how so doing could inform disease control. For instance, they argue, frontline vector‐borne disease prevention tools such as insecticide‐treated bednets and indoor residual spraying rely on reducing mosquito contact rates with human hosts and reducing vector survival. Reduced vector survival has the benefits of decreasing mosquito abundance, the number of bites a mosquito can take over the course of its lifetime, and the probability that mosquitoes survive past the parasite's development time. These effects likely shape the selective environment for parasites within the vector. However, whether parasites can respond to interventions by evolving shorter EIPs or other heritable extended phenotypes that lengthen mosquito survival or change vector behavior merit further investigation.

As in the case of malaria described by Lefevre et al. (2017), life‐history trait evolution theory and its attributes can help better understand the adaptive potential of triatomines—the vector of Chagas disease. The review by Flores‐Ferrer, Marcou, Waleckx, Dumonteil, and Gourbière (2017) suggests that current knowledge of the determinants of high diversity and low virulence of the *Trypanosoma cruzi* parasite remains too limiting to design evolution‐proof strategies, while such attributes may be part of the future of Chagas disease control after the 2020 WHO's target of regional elimination of intradomiciliary transmission has been reached. The authors argue that the eco‐epidemiological relationships that build‐up the selective pressures at work have been assiduously studied over the last century, so that, combined with concepts and modeling inspired from life‐history evolution, a good evolutionary understanding could be rapidly gained. Although more specific information will surely be needed, the authors suggest that an effective research strategy would be to integrate data into the conceptual and theoretical framework of evolutionary ecology and life‐history evolution to provide the quantitative backgrounds necessary to understand and possibly anticipate adaptive responses to public health interventions.

Public health interventions targeting helminth diseases often rely on mass drug administration to reduce human morbidity and mortality. Considering the frequency of such interventions and the strength of the selective pressure they impose, the emergence and spread of drug resistance is a concern. In the case of schistosomiasis, although hotspots of reduced efficacy of the drug praziquantel have been reported, resistance is not widespread. However, parasite populations often exhibit considerable genetic variability in their natural tolerance, or acquired resistance, to drugs which, as Viana, Faust, Haydon, Webster, and Lamberton (2017) emphasize, is related to the fitness costs associated with such resistance compared to susceptible lines. Using Bayesian state‐space models (SSMs) fitted to data from an in vivo laboratory system, the authors tested the hypothesis that the spread of resistant *Schistosoma mansoni* may be limited by life‐history costs not present in susceptible counterparts. *S. mansoni* parasites from a praziquantel‐susceptible (S), a praziquantel‐resistant (R) or a mixed line of originally resistant and susceptible parasites (RS) were exposed to a range of praziquantel doses. Results showed that parasite adult worm survival and fecundity in the murine host decreased across all lines, including R, with increasing drug pressure. The authors also observed trade‐offs between adult survival and fecundity in all untreated lines, and these remained strong in S with praziquantel pressure. In contrast, trade‐offs between adult survival and fecundity were lost under praziquantel pressure in R. Additionally the authors showed that life‐history traits within the molluscan intermediate host were complex, but trade‐offs were demonstrated between parasite establishment and cercarial output. These results have theoretical and applied implications and applications for future schistosomiasis control programs and for other host–parasite treatment programs in general.

Complementing the work by Viana et al., 2017; Borlase, Webster, and Rudge (2017) present the case example of haematobium group *Schistosoma* spp. hybrids in West Africa, a system involving multiple interacting parasites and multiple definitive hosts, in a region where zoonotic reservoirs of schistosomiasis were not previously considered to be of importance. The authors consider how existing mathematical model frameworks for schistosome transmission could be expanded and adapted to zoonotic hybrid systems, exploring how such model frameworks can utilize molecular and epidemiological data, as well as the complexities and challenges this presents. The authors also highlight the opportunities and value such mathematical models could bring to this and a similar multihost, multiparasite systems, including informing priorities for data collection, diagnostics and laboratory studies and exploring the impact that hybridizations may have on control measures, as well the impact that evolutionary pressures including control measures may have on driving the emergence and spread of parasite hybrids.

Evolutionary‐based mathematical modeling is also increasingly used to enhance both understanding and design of integrated intervention in the context of microparasites such as Dengue. Lourenço et al. (2017) review and analyze the biological and epidemiological background of dengue, together with the major achievements of computational approaches including highlighting critical knowledge gaps and research underachievements that call for an urgent renewed focus. The authors argue that possible advancements based on new processing strategies, including real‐time computational analysis of genetic data, phylodynamic modeling frameworks, within‐host model frameworks and GPU accelerated computing already implemented for other pathogens, are already at reach of the Dengue research community. These new approaches are expected to make a significant contribution to our understanding of the evolutionary ecology and immunology of the dengue virus and support the design of novel integrated control strategies adaptable to other microparasite systems such as avian influenza affecting domestic animals and humans alike.

Influenza pandemics represent a significant threat to global public health. Four major pandemics have been recorded since the 1900s, occurring in 1918, 1957, 1968, and 2009 when influenza A viruses with genes from animal sources adapted to the human population, a process known as antigenic shift. The H1N1/2009 pandemic virus emerged from swine that contained gene segments ultimately derived from previously circulating human and avian viruses, highlighting a key role of segmental reassortment of genes from multiple hosts for host adaption and pandemic emergence. In this context, Joseph, Vijaykrishna, Smith, and Su (2017) used a relaxed molecular clock model to test whether the European avian‐like swine (EA‐swine) influenza virus originated through the introduction of a single avian ancestor as an entire genome, followed by an analysis of host‐specific selection pressures among different gene segments. The results indicate independent introduction of gene segments via transmission of avian viruses into swine followed by reassortment events that occurred at least 1–4 years prior to the EA‐swine outbreak. All EA‐swine gene segments exhibited greater selection pressure than avian viruses, reflecting both adaptive pressures and relaxed selective constraints that are associated with host switching. Key amino acid mutations in the viral surface proteins (H1 and N1) that play a role in adaptation to new hosts were also observed suggesting adaptive changes in viral genomes following the transmission of avian influenza viruses to swine and the early establishment of the EA‐swine lineage.

Complementing the work by Joseph et al., 2017; Grear, Hall, Dusek, and Ip (2017) investigated mechanisms of intercontinental highly pathogenic avian influenza virus (HPAIV) spread through wild bird reservoirs possibly related to the North America outbreak in 2014. This introduction resulted in several reassortment events with North American (NA) lineage low‐pathogenic avian influenza viruses and the reassortant EA/NA H5N2 that went on to cause one of the largest HPAIV poultry outbreaks in North America. In their research article, the authors used a time‐rooted phylodynamic model that explicitly incorporated viral population dynamics with evolutionary dynamics to estimate the basic reproductive number (R0) and viral migration among host types in domestic and wild birds, as well as between the EA H5N8 and EA/NA H5N2 in wild birds. While the authors did not find evidence to support the hypothesis that transmission of novel HPAIVs in wild birds was restricted by mechanisms associated with highly pathogenic phenotypes or that the HPAIV poultry outbreak was self‐sustaining and required viral input from wild birds, the model estimates of the transmission parameters suggested that the HPAIV outbreak met or exceeded the threshold for persistence in wild birds (R0 > 1) and poultry (R0 ≈ 1). Overall, the results of this work suggest that this novel HPAIV and reassortments did not encounter any transmission barriers sufficient to prevent persistence when introduced to wild or domestic birds and highlight the relevance of phylodynamic methods to test hypotheses about geographical spread of AIVs in wild birds, multiyear evolutionary processes of AIVs in reservoir hosts and relative fitness of highly pathogenic versus low‐pathogenic AIVs in wild birds for more integrated surveillance systems.

## 3 CLOSING REMARKS: EVOLUTION, ADAPTIVE MANAGEMENT, AND SUSTAINABLE HEALTH DEVELOPMENT

While disease control and prevention have achieved great successes, the paradigm within which it has been developed, including our understanding of host–parasite relationships, infection, and disease, as well as best management practices, arguably is not enough. A major shortcoming is that the solutions it provides, often grounded in the curative domain, are not lasting. Evolutionary thinking applied to medical and public health problems promises to substitute the short‐sighted use of drugs with sustainable solutions: If we cannot eradicate infections by frontal assault, we may be able to keep them at bay durably provided we can understand the fundamental nature of host–parasite relationships.

The evolutionary perspective asks ultimate questions, which are about why mechanisms or epidemiological phenotypes are the way they are (i.e., how has this mechanism given a selective advantage? what is the evolutionary history of this mechanism?). Most medical research and perspectives focus on proximal questions, questions about the mechanisms themselves (i.e., how does the mechanism work, what is the ontogeny of the mechanisms?). The distinction between proximate (mechanistic) and ultimate (evolutionary) explanations was emphasized and formalized several decades ago but remain unfamiliar to the medical sciences and public health domain despite its epistemological importance (Nesse, 2013). Both types of explanations are necessary, neither substitutes for the other, and they inform each other (Nesse, 2013; Nesse & Stearns, 2008). Incorporating evolutionary thinking in infectious disease research helps improving our understanding of diseases transmission dynamics, infection patterns, and disease manifestation trends by superimposing a context‐dependent, systems dynamics prism that appreciates that organisms and their interactions are in constant flux (Levin, 1998). Accordingly, evolutionary biology can help identify new relevant questions such as the ones addressed in this special issue, and doing so, can help inform more integrated disease control interventions as well as more adaptive health management strategies.

Adaptive health management, standing on strong evolutionary foundations, has the potential to address two fundamental errors underpinning most current public health interventions targeting infectious diseases. The first error is often the implicit assumption that pathogens or parasite responses to human intervention and/or that human–parasite/pathogens interactions are linear, predictable, and controllable. The second error is the assumption that pathogens and parasites can be treated independently of the social‐ecological system within which they evolve (Horwitz & Wilcox, 2005). By explicitly avoiding these errors, evolutionary‐based adaptive health management could help strengthen the representation of the operational concept of resilience in public health and restore the health systems capacity to buffer infectious and noninfectious challenges, learn, and develop—as a framework for understanding how to sustain and enhance adaptive capacity in a complex world of rapid transformations (Folke et al., 2002).

## References

[eva12586-bib-0001] Allegranzi, B. , Kilpatrick, C. , Storr, J. , Kelley, E. , Park, B. J. , & Donaldson, L. (2017). Global infection prevention and control priorities 2018–22: A call for action. The Lancet Global Health, 5, e1178–e1180. https://doi.org/10.1016/S2214-109X(17)30427-8 29132606 10.1016/S2214-109X(17)30427-8 PMC7129117

[eva12586-bib-0002] Altizer, S. , Ostfeld, R. S. , Johnson, P. T. J. , Kutz, S. , & Harvell, C. D. (2013). Climate change and infectious diseases: from evidence to a predictive framework. Science, 341, 514–519. https://doi.org/10.1126/science.1239401 23908230 10.1126/science.1239401

[eva12586-bib-0003] Anderson, R. M. , & May, R. M. (1981). The population dynamics of microparasites and their invertebrate hosts. Philosophical Transactions of the Royal Society of London. Series B, Biological sciences, 291, 451–524. https://doi.org/10.1098/rstb.1981.0005 10.1098/rstb.2014.0307 PMC4360116 25750231

[eva12586-bib-0004] Bhatt, S. , Gething, P. W. , Brady, O. J. , Messina, J. P. , Farlow, A. W. , Moyes, C. L. , … Hay, S. I. (2013). The global distribution and burden of dengue. Nature, 496, 504–507. 23563266 10.1038/nature12060 PMC3651993

[eva12586-bib-0005] Birget, P. L. G. , Greischar, M. A. , Reece, S. E. , & Mideo, N. (2017). Altered life history strategies protect malaria parasites against drugs. Evolutionary Application, 11, 442–455. https://doi.org/10.1111/eva.12516 10.1111/eva.12516 PMC5891063 29636798

[eva12586-bib-0006] Borlase, A. , Webster, J. P. , & Rudge, J. W. (2017). Opportunities and challenges for modelling epidemiological and evolutionary dynamics in a multihost, multiparasite system: Zoonotic hybrid schistosomiasis in West Africa. Evolutionary Application, 11, 501–515. https://doi.org/10.1111/eva.12529 10.1111/eva.12529 PMC5891036 29636802

[eva12586-bib-0007] Brady, O. J. , Gething, P. W. , Bhatt, S. , Messina, J. P. , Brownstein, J. S. , Hoen, A. G. , … Hay, S. I. (2012). Refining the global spatial limits of dengue virus transmission by evidence‐based consensus. PLoS Neglected Tropical Diseases, 6, e1760. 22880140 10.1371/journal.pntd.0001760 PMC3413714

[eva12586-bib-0008] Carroll, S. P. , Jørgensen, P. S. , Kinnison, M. T. , Bergstrom, C. T. , Denison, R. F. , Gluckman, P. , … Tabashnik, B. E. (2014). Applying evolutionary biology to address global challenges. Science, 346, 1245993 https://doi.org/10.1126/science.1245993 25213376 10.1126/science.1245993 PMC4245030

[eva12586-bib-0009] Echaubard, P. , Sripa, B. , Mallory, F. F. , & Wilcox, B. A. (2016). The role of evolutionary biology in research and control of liver flukes in Southeast Asia. Infection, Genetics and Evolution, 43, 381–397. https://doi.org/10.1016/j.meegid.2016.05.019 10.1016/j.meegid.2016.05.019 PMC4971136 27197053

[eva12586-bib-0010] Flores‐Ferrer, A. , Marcou, O. , Waleckx, E. , Dumonteil, E. , & Gourbière, S. (2017). Evolutionary ecology of Chagas disease; what do we know and what do we need?. Evolutionary Application, 11, 470–487. https://doi.org/10.1111/eva.12582 10.1111/eva.12582 PMC5891055 29636800

[eva12586-bib-0011] Folke, C. , Carpenter, S. , Elmqvist, T. , Gunderson, L. , Holling, C. S. , & Walker, B. (2002). Resilience and sustainable development: Building adaptive capacity in a world of transformations. AMBIO Journal of the Human Environment, 31, 437–440. https://doi.org/10.1579/0044-7447-31.5.437 10.1579/0044-7447-31.5.437 12374053

[eva12586-bib-0012] Glunt, K. D. , Coetzee, M. , Huijben, S. , Koffi, A. A. , Lynch, P. A. , N'Guessan, R. , … Thomas, M. B. (2017). Empirical and theoretical investigation into the potential impacts of insecticide resistance on the effectiveness of insecticide‐treated bed nets. Evolutionary Application, 11, 431–441. https://doi.org/10.1111/eva.12574 10.1111/eva.12574 PMC5891045 29636797

[eva12586-bib-0013] Grear, D. A. , Hall, J. S. , Dusek, R. J. , & Ip, H. S. (2017). Inferring epidemiologic dynamics from viral evolution: 2014–2015 Eurasian/North American highly pathogenic avian influenza viruses exceed transmission threshold, R0 = 1, in wild birds and poultry in North America. Evolutionary Application, 11, 547–557. https://doi.org/10.1111/eva.12576 10.1111/eva.12576 PMC5891053 29636805

[eva12586-bib-0014] Horwitz, P. , & Wilcox, B. A. (2005). Parasites, ecosystems and sustainability: An ecological and complex systems perspective. International Journal for Parasitology, 35, 725–732. https://doi.org/10.1016/j.ijpara.2005.03.002 15925596 10.1016/j.ijpara.2005.03.002

[eva12586-bib-0015] Huijben, S. , & Paaijmans, K. P. (2017). Putting evolution in elimination: Winning our ongoing battle with evolving malaria mosquitoes and parasites. Evolutionary Application, 11, 415–430. https://doi.org/10.1111/eva.12530 10.1111/eva.12530 PMC5891050 29636796

[eva12586-bib-0016] Joseph, U. , Vijaykrishna, D. , Smith, G. J. D. , & Su, Y. C. F. (2017). Adaptive evolution during the establishment of European avian‐like H1N1 influenza A virus in swine. Evolutionary Applications, 11, 534–546. https://doi.org/10.1111/eva.12536n/a–n/a. 10.1111/eva.12536 PMC5891058 29636804

[eva12586-bib-0040] Kosoy, M. , & Kosoy, R. (2017). Complexity and biosemiotics in evolutionary ecology of zoonotic infectious agents. Evolutionary Applications, 11, 394–403. https://doi.org/10.1111/eva.12503 10.1111/eva.12503 PMC5891042 29636794

[eva12586-bib-0017] Lafferty, K. D. (2009). The ecology of climate change and infectious diseases. Ecology, 90, 888–900. https://doi.org/10.1890/08-0079.1 19449681 10.1890/08-0079.1

[eva12586-bib-0018] Lederberg, J. , Shope, R. E. , & Oaks, S. C. (1992). Emerging Infections: Microbial Threats to Health in the United States. Washington: National Academies Press. 25121245

[eva12586-bib-0019] Lefevre, T. , Ohm, J. , Dabiré, K. R. , Cohuet, A. , Choisy, M. , Thomas, M. B. , & Cator, L. (2017). Transmission traits of malaria parasites within the mosquito: Genetic variation, phenotypic plasticity, and consequences for control. Evolutionary Application, 11, 456–469. https://doi.org/10.1111/eva.12571 10.1111/eva.12571 PMC5891056 29636799

[eva12586-bib-0020] Levin, S. A. (1998). Ecosystems and the biosphere as Complex Adaptive Systems. Ecosystems, 1, 431–436. https://doi.org/10.1007/s100219900037

[eva12586-bib-0021] Lourenço, J. , Tennant, W. , Faria, N. R. , Walker, A. , Gupta, S. , & Recker, M. (2017). Challenges in dengue research: A computational perspective. Evolutionary Application, 11, 516–533. https://doi.org/10.1111/eva.12554 10.1111/eva.12554 PMC5891037 29636803

[eva12586-bib-0022] McMichael, A. J. , & Wilcox, B. A. (2009). Climate Change, Human Health, and Integrative Research: A Transformative Imperative. EcoHealth, 6, 163–164. https://doi.org/10.1007/s10393-009-0262-9 20082115 10.1007/s10393-009-0262-9

[eva12586-bib-0023] McNeill, W. H. (1976). Plagues and peoples. New York: Anchor Press.

[eva12586-bib-0024] Myers, S. S. , Gaffikin, L. , Golden, C. D. , Ostfeld, R. S. , Redford, H. , Ricketts, K. H. T. , … Osofsky, S. A. (2013). Human health impacts of ecosystem alteration. Proceedings of the National Academy of Sciences of the United States of America, 110, 18753–18760. https://doi.org/10.1073/pnas.1218656110 24218556 10.1073/pnas.1218656110 PMC3839693

[eva12586-bib-0025] Myers, S. S. , & Patz, J. A. (2009). Emerging Threats to Human Health from Global Environmental Change. Annual Review of Environment and Resources, 34, 223–252. https://doi.org/10.1146/annurev.environ.033108.102650

[eva12586-bib-0026] Nesse, R. (2008). What evolutionary biology offers public health. Bulletin of the World Health Organization, 86, 83 https://doi.org/10.2471/BLT.00.00000 18297156 10.2471/BLT.07.049601 PMC2647384

[eva12586-bib-0027] Nesse, R. M. (2013). Tinbergen's four questions, organized: A response to Bateson and Laland. Trends in Ecology & Evolution, 28, 681–682. https://doi.org/10.1016/j.tree.2013.10.008 24216179 10.1016/j.tree.2013.10.008

[eva12586-bib-0028] Nesse, R. M. , & Stearns, S. C. (2008). The great opportunity: Evolutionary applications to medicine and public health: Evolutionary applications to medicine and public health. Evolutionary Applications, 1, 28–48. https://doi.org/10.1111/j.1752-4571.2007.00006.x 25567489 10.1111/j.1752-4571.2007.00006.x PMC3352398

[eva12586-bib-0029] Packard, R. M. (2007). The making of a tropical disease: A short history of malaria. Baltimore, MD, USA: JHU Press.

[eva12586-bib-0030] Restif, O. (2009). Evolutionary epidemiology 20 years on: Challenges and prospects. Infection, Genetics and Evolution, 9, 108–123. https://doi.org/10.1016/j.meegid.2008.09.007 10.1016/j.meegid.2008.09.007 18977460

[eva12586-bib-0031] Stearns, S. C. , & Koella, J. C. (2008). Evolution in Health and Disease, 2nd edn Oxford , New York: Oxford University Press.

[eva12586-bib-0032] Sternberg, E. D. , & Thomas, M. B. (2017). Insights from agriculture for the management of insecticide resistance in disease vectors. Evolutionary Application, 11, 404–414. https://doi.org/10.1111/eva.12501 10.1111/eva.12501 PMC5891047 29636795

[eva12586-bib-0033] Tarleton, R. L. , Reithinger, R. , Urbina, J. A. , Kitron, U. , & Gürtler, R. E. (2007). The challenges of Chagas disease— grim outlook or glimmer of hope? PLoS Medicine, 4, e332. 18162039 10.1371/journal.pmed.0040332 PMC2222930

[eva12586-bib-0034] Valen, V. (1973). A new evolutionary law. Evolutionary Theory, 1, 1–30.

[eva12586-bib-0035] Viana, M. , Faust, C. L. , Haydon, D. T. , Webster, J. P. , & Lamberton, P. H. L. (2017). The effects of subcurative praziquantel treatment on life‐history traits and trade‐offs in drug‐resistant *Schistosoma mansoni* . Evolutionary Application, 11, 488–500. https://doi.org/10.1111/eva.12558 10.1111/eva.12558 PMC5891057 29636801

[eva12586-bib-0036] Whitmee, S. , Haines, A. , Beyrer, C. , Boltz, F. , Capon, A. G. , de Dias, B. F. , … Yach, D. (2015). Safeguarding human health in the Anthropocene epoch: Report of The Rockefeller Foundation‐Lancet Commission on planetary health. The Lancet, 386, 1973–2028. https://doi.org/10.1016/S0140-6736(15)60901-1 10.1016/S0140-6736(15)60901-1 26188744

[eva12586-bib-0037] WHO . (2016). World malaria report: 2016.

[eva12586-bib-0038] Wilcox, B. A. , & Echaubard, P. (2016). Balancing biomedical and ecological perspectives in research framing of liver fluke and cholangiocarcinoma in NE Thailand. Parasitology International, 66, 372–377. https://doi.org/10.1016/j.parint.2016.10.002 27729246 10.1016/j.parint.2016.10.002

[eva12586-bib-0039] World Health Organization & UNICEF . (2004). Prevention and control of schistosomiasis and soil‐transmitted helminthiasis. Geneva: World Health Organization. 12592987

